# Three-dimensional collagen matrix induces a mechanosensitive invasive epithelial phenotype

**DOI:** 10.1038/srep42088

**Published:** 2017-02-10

**Authors:** Shawn P. Carey, Karen E. Martin, Cynthia A. Reinhart-King

**Affiliations:** 1 Department of Biomedical Engineering, Cornell University, Ithaca, New York 14853, USA

## Abstract

A critical step in breast cancer progression is local tissue invasion, during which cells pass from the epithelial compartment to the stromal compartment. We recently showed that malignant leader cells can promote the invasion of otherwise non-invasive epithelial follower cells, but the effects of this induced-invasion phenomenon on follower cell phenotype remain unclear. Notably, this process can expose epithelial cells to the stromal extracellular matrix (ECM), which is distinct from the ECM within the normal epithelial microenvironment. Here, we used a 3D epithelial morphogenesis model in which cells were cultured in biochemically and mechanically defined matrices to examine matrix-mediated gene expression and the associated phenotypic response. We found that 3D collagen matrix promoted expression of mesenchymal genes including MT1-MMP, which was required for collagen-stimulated invasive behavior. Epithelial invasion required matrix anchorage as well as signaling through Src, PI3K, and Rac1, and increasingly stiff collagen promoted dispersive epithelial cell invasion. These results suggest that leader cell-facilitated access to the stromal ECM may trigger an invasive phenotype in follower epithelial cells that could enable them to actively participate in local tissue invasion.

Local tissue invasion is a key transition in solid tumor progression toward metastatic disease during which cells from the epithelial compartment bypass the basement membrane and cross into the underlying interstitial stroma^1^. Since invasion involves suppression of normal homeostatic epithelial behaviors and orchestration of extracellular matrix (ECM) remodeling and cell motility programs, this process is mechanistically burdensome^2^, and it is unlikely that all of the cells within a tumor are invasion-competent^3^. Notably, it has been suggested that cooperation among distinct cellular subtypes within the tumor microenvironment could facilitate several elements of cancer progression, including invasion and metastasis^4^,^5^. Our group and others have provided experimental evidence for this notion, showing that extracellular matrix remodeling by invasive malignant cells or stromal fibroblasts can induce a co-invasive phenotype by which otherwise non-invasive epithelial cells can enter and migrate through the stromal ECM^6^,^7^,^8^. During this process, proteolytic ECM patterning by leader cells can result in the formation of matrix microtracks that provide physical space to enable unimpeded migration by follower cells^9^,^10^,^11^. However, the comprehensive effects of the induced-invasion phenomenon on follower cell phenotype remain to be determined, and it is unclear how escape from the protective epithelial compartment and transit into the stromal compartment affects nontransformed epithelial cells.

Among the most significant differences between the epithelial and stromal tissue compartments is the distinct extracellular matrix that comprises each^12^. Whereas epithelial basement membrane is a thin, dense meshwork primarily consisting of laminin and type IV collagen^13^, the interstitial stromal ECM is a structurally heterogeneous fibrillar network dominated by type I collagen^14^. In developing, homeostatic, and diseased mammary tissue, basement membrane and interstitial ECM biochemistry, architecture, and mechanics are key regulators of epithelial cell phenotype^12^,^15^, acting primarily through ECM-specific integrin-based adhesion and signaling^16^,^17^,^18^,^19^,^20^. Critically, even during extensive physiological tissue remodeling, hyperplastic disorders, and carcinoma *in situ*, epithelial/carcinoma cells remain adherent to the epithelial basement membrane, only accessing the type I collagen-rich stroma during tumor invasion^1^.

To model the effects of mammary epithelial cell exposure to stromal type I collagen ECM, we utilized an *in vitro* epithelial morphogenesis model. This strategy was not intended to model physiological epithelial branching morphogenesis or pathological matrix-directed disease progression as previously described^21^,^22^,^23^,^24^,^25^. Rather, we used this model to provide a simple 3D culture system with which to simultaneously examine the resulting collagen matrix-directed epithelial cell phenotype and the underlying collagen matrix-mediated gene expression. We found that, compared to culture in 3D basement membrane (Matrigel), 3D type I collagen matrix induced mesenchymal gene expression and promoted an MT1-MMP-dependent invasive epithelial phenotype that was driven by protrusive signaling and sensitive to collagen ECM structure and mechanics.

## Results

### Three-dimensional collagen induces an invasive epithelial phenotype

**Figure 1:**
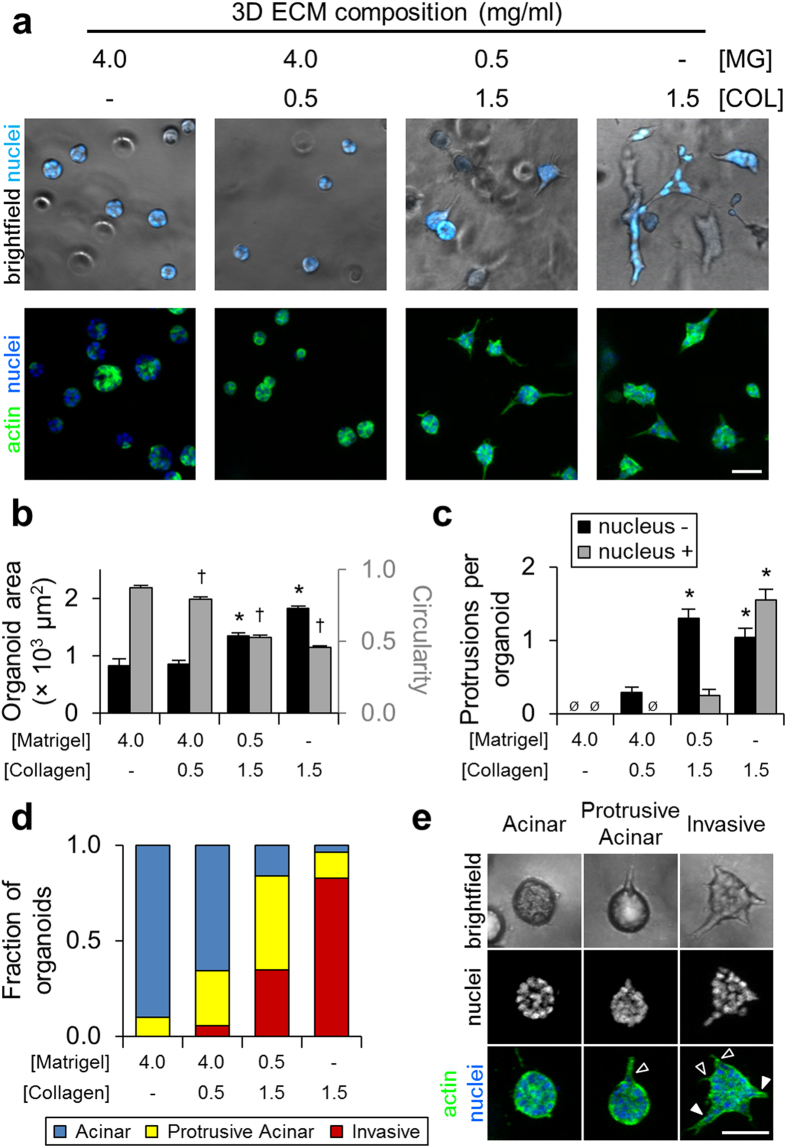
3D matrix-induced epithelial cell phenotypes. (**a**) Brightfield and fluorescent images of MCF-10A epithelial organoids after 4 days of growth in the indicated ECM. (**b**) Quantification of organoid area (black bars) and circularity (grey bars). *P < 0.001 as compared to Matrigel area; ^†^P < 0.001 as compared to Matrigel circularity. (**c**) Quantification of nucleus-free (‘nucleus −’, black bars) and nucleus-containing protrusions (‘nucleus +’, grey bars) exhibited by epithelial organoids. ^Ø^none detected. *P < 0.05 as compared to no observed protrusions in Matrigel. Data in (**b**,**c**) are mean ± s.e.m. from n > 100 organoids per condition from 3 independent experiments. (**d**) Quantification of organoid morphology for n > 100 organoids per condition from 3 independent experiments. Circularity was used to determine organoid morphology as described in the text. (**e**) Representative images of acinar, protrusive acinar, and invasive MCF-10A epithelial organoids. Protrusive acinar and invasive organoids exhibit nucleus-free (black arrowheads) and nucleus-containing (white arrowhead) protrusions. Scale bars: 50 μm.

To determine the effect of matrix composition on mammary epithelial phenotype, we used a 3D morphogenesis assay in which Matrigel and type I collagen represented the basement membrane and stromal extracellular matrix, respectively. We selected the mammary cell line MCF-10A as a model for normal epithelial cells in this study. Although these cells are more basal-like than cells from normal breast tissue, they are non-tumorigenic *in vivo* and possess many characteristics of normal mammary epithelial cells^26^,^27^. After 4 days of 3D culture, single MCF-10A epithelial cells proliferated to form multicellular clusters of cells, or organoids, whose morphologies were matrix-dependent (Fig. 1a). Cells in pure Matrigel formed acinar organoids consistent with previous studies^28^, and as collagen content was increased and Matrigel content decreased, organoids became increasingly invasive, losing their rounded morphology and becoming stellate and protrusive. We quantified matrix-directed morphological changes to find that organoids in collagen were significantly larger and showed decreased circularity (Fig. 1b), indicative of increased protrusivity. Whereas organoids in Matrigel and collagen-supplemented Matrigel showed no significant protrusions, organoids in Matrigel-supplemented collagen exhibited 1.3 ± 0.1 nucleus-free cytoplasmic protrusions, and organoids in pure collagen matrix uniquely generated both nucleus-free (1.0 ± 0.1 per organoid) and nucleus-containing (1.6 ± 0.1 per organoid) protrusions (Fig. 1c). We measured the circularity of multicellular structures to categorize organoids as acinar (circularity > 0.8), protrusive acinar (circularity 0.6–0.8), and invasive (circularity < 0.6) and found that increasing collagen content and decreasing Matrigel suppressed acinar morphologies and promoted an invasive phenotype (Fig. 1d). Overall, acinar structures were rounded and exhibited no protrusive extensions, protrusive acinar organoids were rounded and typically contained one or more nucleus-free protrusion (Fig. 1e; black arrowhead), and invasive organoids were morphologically heterogeneous and generally contained one or more nucleus-free and nucleus-containing protrusion (Fig. 1e; black and white arrowheads, respectively). Together, these results confirmed that the 3D collagen-induced invasive phenotype involves expansive outgrowth, protrusion, and cell body translation into the surrounding ECM.

### Three-dimensional collagen promotes mesenchymal gene expression

**Figure 2:**
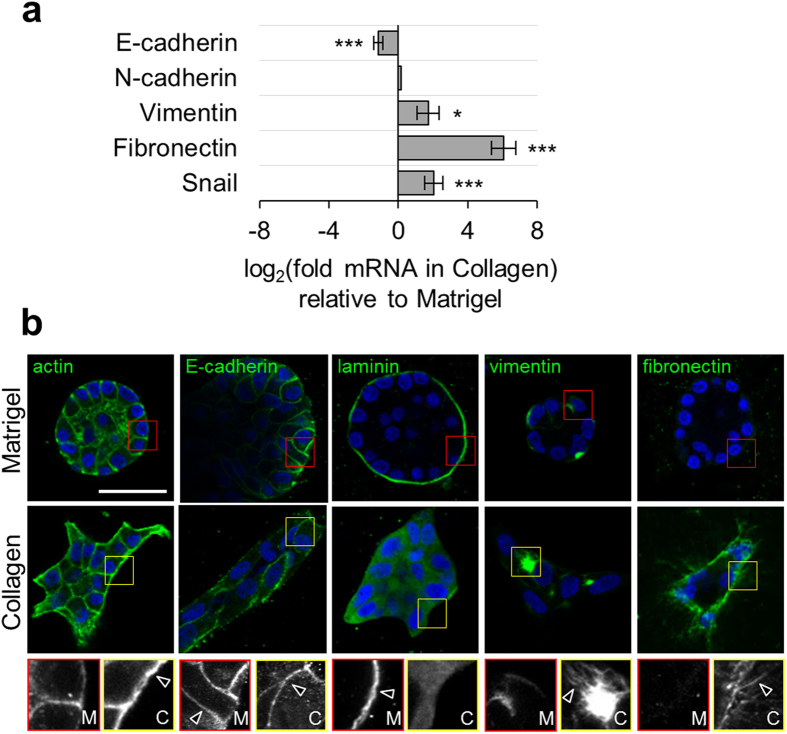
3D matrix-dependent gene expression. (**a**) Gene expression of MCF-10A in 1.5 mg/ml collagen and 4.0 mg/ml Matrigel measured by quantitative real-time RT-PCR. Data are represented as log_2_(collagen expression/Matrigel expression) such that positive values indicate upregulation and negative values indicate downregulation relative to Matrigel. Data are mean ± s.e.m. from at least 3 independent experiments. *P = 0.01; ***P < 0.0001. (**b**) Representative confocal images of actin, E-cadherin, laminin, vimentin, and fibronectin staining in organoids developed in Matrigel and collagen. Insets and arrowheads highlight characteristic features in Matrigel (red) and collagen (yellow). Scale bar: 50 μm.

To better understand how the ECM composition regulates epithelial cell behavior, we measured 3D matrix-dependent gene expression using quantitative real-time RT-PCR and indirect immunofluorescence. We found that collagen downregulates E-cadherin expression while inducing significant upregulation of the mesenchymal markers vimentin, fibronectin, and Snail (Fig. 2a). With the exception of E-cadherin, which showed prominent localization at cell-cell junctions in both Matrigel and collagen matrices, we observed striking matrix-dependent differences in protein expression and localization by confocal imaging (Fig. 2b; insets and arrowheads). F-actin was abundant and formed continuous trans-cellular cables around the periphery of invasive structures in collagen matrix, but only weak cortical staining in acinar organoids formed in Matrigel. Laminin was deposited in a continuous basement membrane surrounding organoids in Matrigel, but not collagen. The intermediate filament vimentin showed considerable cell-to-cell variability in both Matrigel and collagen, but uniquely organized into filamentous networks in invasive organoids formed in collagen. Finally, whereas acinar organoids in Matrigel showed no fibronectin expression by immunofluorescence, invasive organoids in collagen matrix showed extensive extracellular deposition of fibrillar fibronectin. Together, these data indicate that the 3D collagen microenvironment promotes mesenchymal gene and protein expression.

### 3D collagen-induced MT1-MMP expression and activity is required for epithelial invasion through restrictive ECM

**Figure 3:**
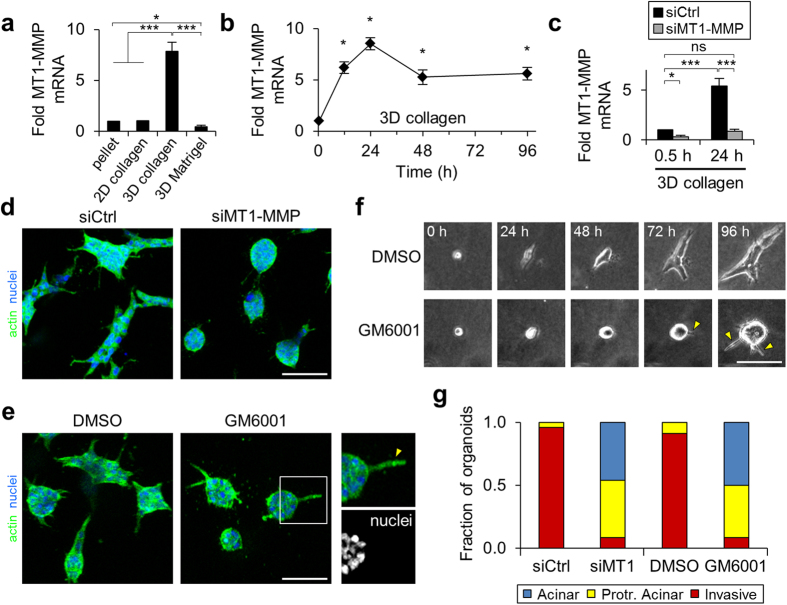
Regulation of invasive epithelial phenotype by matrix-induced MT1-MMP. (**a**) MT1-MMP gene expression by MCF-10A epithelial cells following 24 h of culture on 2D collagen-coated glass (0.1 mg/ml), in 3D collagen matrix (1.5 mg/mL), or in 3D Matrigel matrix (4.0 mg/mL). Data are normalized to cell pellet lysed prior to seeding and represented as mean ± s.e.m. from at least 3 independent experiments. *P = 0.04; ***P < 0.0001. (**b**) MT1-MMP expression during 96 h of culture in 3D collagen matrix. Data are represented as mean ± s.e.m. from at least 3 independent experiments. *P < 0.001 compared to 0.5 h. (**c**) MT1-MMP expression in cells treated with 1 nM MT1-MMP targeting or non-targeting control siRNA and seeded in 3D collagen for 0.5 h and 24 h. Data are represented as mean ± s.e.m. from at least 3 independent experiments. *P = 0.02; ***P = 0.002; ns, not significant. (**d**) siRNA-treated epithelial organoids after 4 days of 3D collagen culture. Scale bar: 100 μm. (**e**) Epithelial organoids after 4 days of 3D collagen culture treated with GM6001 (0.1 μM) or equivalent DMSO control. Inset and yellow arrowhead indicate nucleus-free protrusion characteristic of protrusive acinar organoids with MT1-MMP-targeting siRNA and GM6001 treatment. Scale bar: 100 μm. (**f**) Time-lapse image series of epithelial morphogenesis in 3D collagen matrix with GM6001 or DMSO control treatment. Yellow arrowheads indicate nucleus-free protrusions into the surrounding matrix with GM6001 treatment. Scale bar: 50 μm. (**g**) Quantification of organoid morphology for n > 50 organoids per treatment from 2–3 independent experiments.

Since membrane type 1 matrix metalloproteinase (MT1-MMP) is the primary enzyme used by stromal and cancer cells to degrade and migrate through fibrillar collagen^29^,^30^ and the invasive epithelial phenotype involved cells moving outward through the surrounding collagen matrix, we next investigated whether MT1-MMP contributes to epithelial invasion in this system. We found that cells in 3D collagen matrix uniquely upregulated MT1-MMP expression, whereas cells in 3D Matrigel significantly downregulated MT1-MMP and cells seeded on 2D collagen-coated glass showed no change in MT1-MMP expression after 24 h (Fig. 3a). Elevated MT1-MMP expression in 3D collagen matrix was maintained throughout the 96-h experiment (Fig. 3b). To determine if MT1-MMP was responsible for invasive behavior, we knocked down MT1-MMP prior to cell seeding in collagen using siRNA. Treatment with 1 nM targeting siRNA significantly reduced MT1-MMP expression to ~30% of non-targeting siRNA levels in cell pellets prior to seeding and prevented collagen-induced upregulation of MT1-MMP at 24 h (Fig. 3c). Notably, MT1-MMP knockdown suppressed epithelial invasion, reverting organoids to more acinar and protrusive acinar morphologies (Fig. 3d). Since previous work has indicated MT1-MMP has critical non-proteolytic functions during epithelial branching morphogenesis^31^, we tested whether the invasive epithelial phenotype we observed here was dependent on MMP activity by treating cells with the broad spectrum MMP inhibitor GM6001. Even low level GM6001 (0.1 μM) treatment suppressed invasive outgrowth of epithelial cells, indicating the proteolytic activity is required for epithelial invasion in our system (Fig. 3e). Both MT1-MMP knockdown and GM6001 treatment induced long, actin-rich anuclear cell extensions at the periphery of some organoids (Fig. 3e; inset and arrowhead). Of note, the non-invasive structures observed under MMP inhibition did not show the acellular core characteristic of MCF-10A organoids grown within Matrigel (Fig. 2b), but rather showed a dense core of nuclei with no (“acinar”) or one or more (“protrusive acinar”) anuclear protrusions into the surrounding ECM. Time-lapse imaging of organoid formation under DMSO vehicle treatment showed outward protrusion of cell bodies as epithelial cells proliferated, resulting in invasive multicellular structures by day 4 (Fig. 3f). GM6001 treatment prevented this outward cell movement during proliferation and limited protrusion into the surround matrix to long, cytoplasmic extensions (Fig. 3f; arrowheads). Therefore, whereas cells treated with control siRNA and DMSO vehicle formed primarily invasive organoids, both MT1-MMP knockdown and GM6001 treatment resulted in formation of acinar and protrusive acinar epithelial organoids (Fig. 3g).

**Figure 4:**
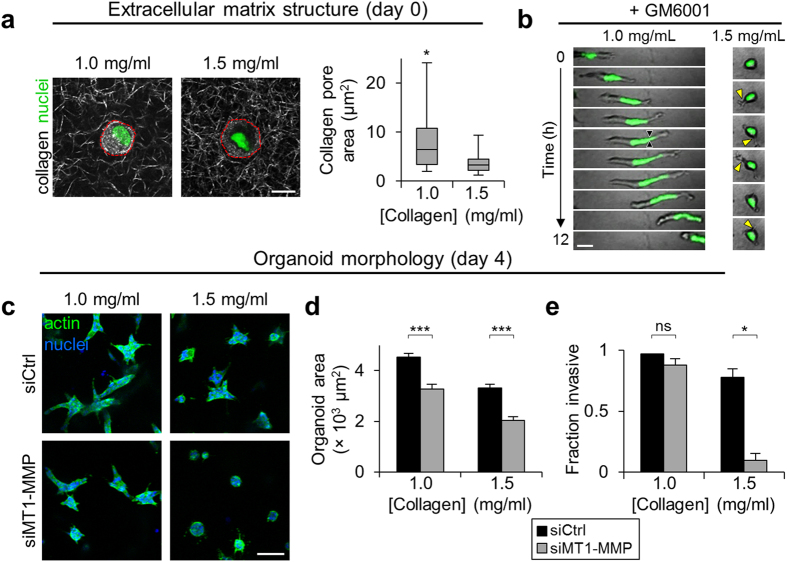
Collagen matrix pore size-dependent epithelial cell migration and invasion. (**a**) Confocal fluorescence and reflectance images of MCF-10A/H2B-GFP cells in 1.0 mg/ml and 1.5 mg/ml collagen matrices demonstrating representative cell nuclei (green) and collagen structure (grey). Dashed red lines indicate cell membrane of rounded cells. Scale bar: 10 μm. Quantification of collagen matrix pore cross-sectional area from 2 independent experiments. *P < 0.0001. (**b**) Time-lapse image series of single MCF-10A/H2B-GFP cells treated with GM6001 (0.1 μM) migrating in 1.0 mg/ml and 1.5 mg/ml collagen matrices. Black arrowheads indicate nucleus constriction site during MMP-independent migration in low density (large pore) matrix. Yellow arrowheads indicate transient cytoplasmic protrusions characteristic of MMP-inhibited cells in high density (small pore) matrix. Scale bar: 25 μm. (**c**) siRNA-treated (1 nM) epithelial organoids after 4 days of 3D culture in 1.0 mg/ml and 1.5 mg/ml collagen matrices. Scale bar: 100 μm. (**d**) Epithelial organoid area. Data are represented as mean ± s.e.m. for n > 100 organoids per condition from 2 independent experiments. ***P < 0.0001. (**e**) Fraction of invasive epithelial organoids. Data are represented as mean ± s.e.m. for n > 100 organoids from 2 independent experiments. *P = 0.01; ns, not significant.

The requirement for MMP activity during mesenchymal cell migration through three-dimensional matrix is dependent on matrix pore size, with larger pores permitting MMP-independent movement^32^. Therefore, we tested whether the requirement for MMP activity in epithelial invasion was similarly sensitive to matrix structure. We seeded cells in 1.0 mg/ml in addition to 1.5 mg/ml collagen matrix and used confocal reflectance microscopy to image the collagen ECM surrounding cells following matrix polymerization. As expected, the distributions of pore sizes measured from single confocal reflectance slices were broad, and reducing collagen density significantly increased pore size from ~1–10 μm^2^ in 1.5 mg/ml collagen matrix to ~1–25 μm^2^ in 1.0 mg/ml collagen matrix (Fig. 4a). Time-lapse imaging showed that GM6001 treatment prevented cell migration in higher density matrix, where cells were unable to translocate their nucleus and continually extended thin cytoplasmic protrusions into the surrounding matrix (Fig. 4b; yellow arrowheads). Conversely, GM6001-treated cells in low density matrix retained their ability to migrate and notably showed transient pauses in migration during which the nucleus arrested and deformed (Fig. 4b; black arrowheads), consistent with nuclear deformation through matrix pores during protease-independent 3D migration^32^,^33^. The requirement for MT1-MMP during epithelial organoid morphogenesis was also dependent on collagen matrix structure (Fig. 4c). Whereas MT1-MMP knockdown induced a small but significant decrease in organoid area in both matrix densities (Fig. 4d), it caused a dramatic decrease in the fraction of organoids showing an invasive phenotype only in higher density 1.5 mg/ml matrix (Fig. 4e). Together, these results indicate that 3D collagen induces upregulation of MT1-MMP, the activity of which is required for epithelial invasion through restrictive 3D matrix but dispensable for epithelial cell movement through large pores in lower density matrix.

### Invasive epithelial phenotype is driven by protrusive signaling

**Figure 5:**
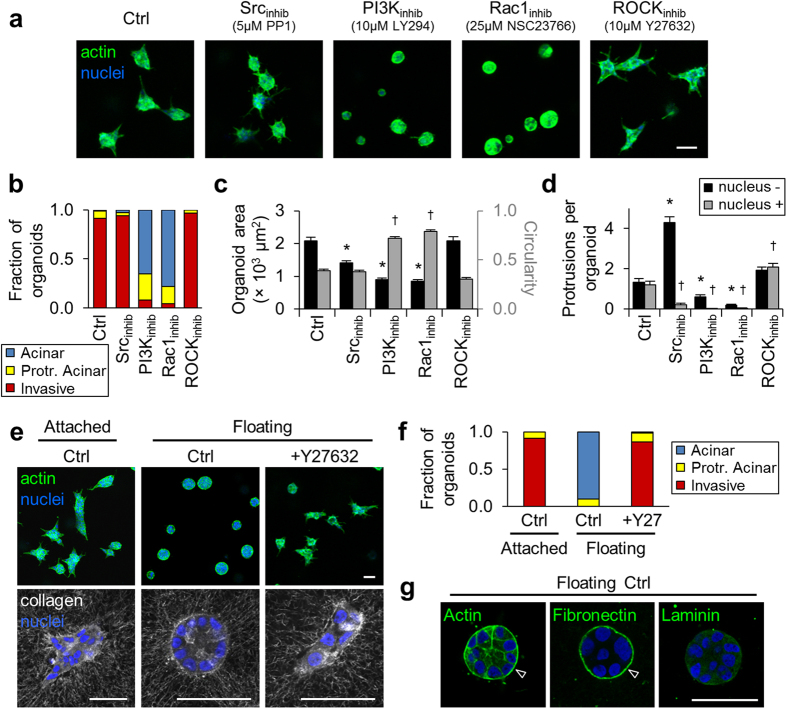
Molecular regulation of the invasive epithelial phenotype. (**a**) MCF-10A epithelial organoids after 4 days of culture in 1.5 mg/ml collagen treated as indicated. (**b**) Quantification of organoid morphology for n > 50 organoids per treatment from 2 independent experiments. (**c**) Quantification of organoid area (black bars) and circularity (grey bars). *P < 0.0001 as compared to Ctrl area; ^†^P < 0.0001 as compared to Ctrl circularity. (**e**) Quantification of ‘nucleus −’ (black bars) and ‘nucleus +’ (grey bars) protrusions exhibited by epithelial organoids. *P < 0.0001 as compared to Ctrl ‘nucleus −’; ^†^P < 0.0001 as compared to Ctrl ‘nucleus +’. Data in (**c**,**d**) are mean ± s.e.m. from n > 50 organoids per treatment from 2 independent experiments. (**e**) Top panels: Epithelial organoids formed in attached and floating collagen matrices. Bottom panels: Confocal reflectance images of collagen matrix (grey) surrounding organoids. (**f**) Quantification of organoid morphology for n > 60 organoids per condition from 3 independent experiments. (**g**) Representative images of actin, fibronectin, and laminin localization in organoids formed in floating collagen matrices. Arrowheads indicate strong localization of actin and fibronectin at the organoid periphery. Scale bars: 50 μm.

We next sought to identify molecular regulators of the MT1-MMP-dependent collagen-induced invasive epithelial phenotype. Since type I collagen can influence epithelial cell behavior through adhesion signaling and the Rho family of GTPases^34^,^35^,^36^,^37^, we examined mammary epithelial morphogenesis under pharmacological inhibition of these major signaling pathways. Inhibition of Src, PI3K, Rac1, and ROCK activity elicited diverse changes in epithelial morphogenesis that altered organoid morphology (Fig. 5a). Whereas control, Src-inhibited, and ROCK-inhibited cells primarily formed invasive structures, PI3K and Rac1 inhibition suppressed epithelial invasion, resulting in acinar and protrusive acinar organoids (Fig. 5b). Inhibition of Src, PI3K, and Rac1 activity significantly reduced organoid size, and PI3K and Rac1 inhibition increased organoid circularity (Fig. 5c), indicative of the more rounded acinar structures formed with these treatments. Notably, Src inhibition significantly reduced nucleus-containing protrusion and increased nucleus-free protrusion (Fig. 5d), mimicking the MMP-inhibited protrusive acinar phenotype. Thus, while the enhanced formation of anuclear protrusions with Src inhibition decreased circularity to cause an apparent increase in organoid invasiveness (Fig. 5b), Src inhibition, as well as PI3K and Rac1 inhibition, effectively prevented collagen-induced cellular outgrowth from epithelial organoids.

Interestingly, the only measured effect of ROCK inhibition on the invasive epithelial phenotype was an increase in the number of nucleus-containing protrusions per organoid, suggesting that ROCK activity is not required for invasive outgrowth and may actually act as an invasion suppressor in this system. Since ROCK-mediated cell contractility plays critical functions in both 3D cell migration and detection of 3D matrix properties^11^,^32^,^34^, this finding led us to question the role of ECM mechanics in regulating the collagen-induced invasive epithelial phenotype. We found that epithelial invasion was prevented by floating the collagen matrix following polymerization to release intra-matrix tension (Fig. 5e; top panels). However, ROCK inhibition rescued the invasive phenotype in floating matrix (Fig. 5e,f), further supporting the hypothesis that ROCK activity suppresses epithelial invasion in this model. Notably, the phenotypic conversion to acinar organoid morphology in floating collagen matrix was not associated with a complete reversion to Matrigel-derived epithelial acini, although some structures with acellular interiors were observed in both conditions. Similar to invasive organoids in attached collagen matrix and distinct from non-invasive acinar organoids in Matrigel (Fig. 2b), rounded organoids in floating collagen showed strong peripheral staining of actin and deposited fibronectin, but not laminin (Fig. 5g; arrowheads). Interestingly, both invasive and acinar organoids in attached and floating matrix, respectively, showed radial alignment and increased local density of collagen fibers at the organoid periphery (Fig. 5e; bottom panels). ROCK inhibition prevented this fiber realignment and condensation, indicating that local matrix remodeling is ROCK-dependent, but is neither required nor sufficient for invasion. Taken together, these results provide evidence that the collagen-induced invasive epithelial phenotype requires intra-matrix tension and is driven by protrusive signaling rather than cell contractility and matrix remodeling.

### Stiffening of collagen ECM promotes epithelial dispersion

**Figure 6:**
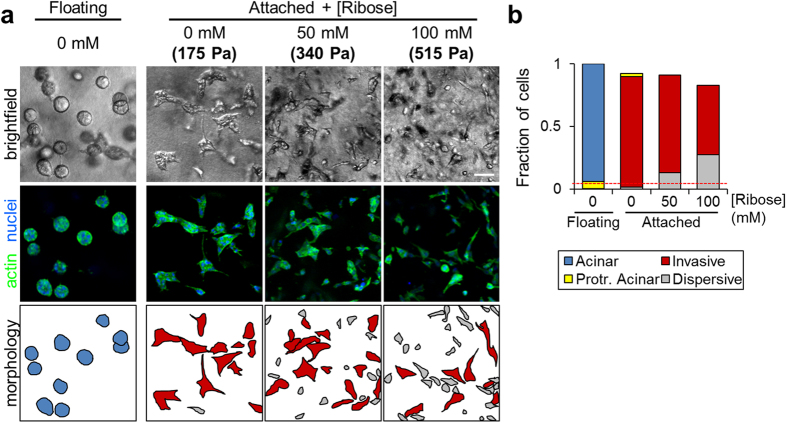
Mechanosensitivity of the invasive epithelial phenotype. (**a**) MCF-10A epithelial organoids after 4 days of culture in floating collagen matrix and attached collagen matrix of increasing stiffness. Brightfield, fluorescence, and cartoon representation of cell/organoid morphologies. Cell/organoid cartoon morphologies are color-coded according to legend in (**b**). Scale bar: 100 μm. (**b**) Quantification of relative cell density and cell organization as single cells (grey bars) or into multicellular organoids (black bars) for n > 700 cells per condition from 2 independent experiments. Dashed red line indicates initial cell density at seeding.

The requirement for attached collagen matrix indicated that the invasive epithelial phenotype is mechanosensitive. While this finding is itself interesting, we sought to investigate this further by simulating the effects of increasingly stiff stromal extracellular matrix that has been associated with tumor progression^23^ and examining the behavior of MCF-10A cells cultured in collagen matrix of increasing stiffness. As previously reported, collagen matrix was stiffened via non-enzymatic glycation by incubating acidified collagen solution with varying amounts of ribose prior to cell embedding, resulting in final compressive moduli of 175 Pa (0 mM), 340 Pa (50 mM), and 515 Pa (100 mM)^38^. Examining epithelial collagen cultures after 4 days, we found that 3D epithelial phenotype was dependent on the mechanical properties of the ECM (Fig. 6a). Whereas all cells were part of multicellular organoids in floating collagen matrices, stiffening of attached collagen matrix increased the fraction of dispersed single epithelial cells (Fig. 6b). Although increasing matrix stiffness caused a modest reduction in overall cell density, presumably due to changes in growth kinetics within stiffer matrices, this effect was not large enough to account for the abundance of single cells in glycated collagen after 4 days since the initial seeding density was equivalent to ~5% of the final cell count in floating collagen matrices (Fig. 6b; dashed red line). Thus, both epithelial cells’ ability to form multicellular structures and the nature of multicellular organoid structures were dependent upon 3D collagen matrix mechanics (Fig. 6b). Together, these data suggest that epithelial cell exposure to increasingly stiff collagen ECM converts the cohesive invasive epithelial phenotype to a dispersive invasive phenotype.

## Discussion

The infiltration of cells from the epithelial compartment into the stromal extracellular matrix is a critical event in tumor progression that serves as an important clinical indicator and determinant of management strategy^2^,^39^. Motivated by recent findings that malignant epithelial and stromal cells can act as path-making leader cells to induce stromal invasion of otherwise invasion-incompetent cells^6^,^7^,^8^, the focus of the current study was to examine the effect of exposure to stromal type I collagen on 3D mammary epithelial cell behavior to determine if access to the stromal compartment during invasion is sufficient to induce an aberrant epithelial phenotype in the absence of cell transformation. We demonstrate that culture in 3D collagen matrix induces expression of mesenchymal genes including fibronectin and vimentin, as well as MT1-MMP, which is required for protrusive outgrowth of epithelial cells through matrix pores in the surrounding collagen ECM. Protrusive signaling and collagen matrix mechanics serve as determinants of the collagen-induced invasive epithelial phenotype. These results suggest that escape from the protective basement membrane-enveloped epithelial compartment and transit into the collagen-rich stromal microenvironment could induce an invasive, mesenchymal-type phenotype in epithelial cells.

The epithelial microenvironment is widely appreciated as a fundamental regulator of mammary cell phenotype during development, functional differentiation, the deregulation of nontransformed epithelium, and invasive cell migration^40^,^41^,^42^,^43^. We show here that exposure of nontransformed mammary epithelial cells to type I collagen ECM induces expression of the mesenchymal genes vimentin, fibronectin, Snail, and MT1-MMP relative to culture in 3D Matrigel. Although the existence of a complete epithelial-mesenchymal transition (EMT) in cancer progression is controversial^44^, there is evidence that the cellular microenvironment can promote mesenchymal gene expression and phenotypes in epithelial cells through growth factor signaling as well as ECM mechanics and composition^16^,^45^,^46^,^47^,^48^. The ECM-mediated mesenchymal shift and enhanced invasiveness we observe here are consistent with previous work showing that both fibronectin and type I collagen induce mesenchymal gene expression in epithelial cells^48^,^49^,^50^, which increases mammary epithelial cell cytoskeletal dynamics and migration^51^. Although ECM properties could modulate growth factor signaling to elicit these changes^40^,^50^,^52^, there is evidence that type I collagen promotes mesenchymal traits directly via β1-integrin^16^ and PI3K-Rac1-JNK signaling^36^. Furthermore, β1-integrin can activate PI3K to enhance Rac1 activity^35^, and we found here that the characteristic induction of protrusive behavior by 3D collagen matrix is PI3K- and Rac1-dependent. Interestingly, both type I collagen and aberrant PI3K-Rac1 activity disrupt mammary epithelial polarity^35^,^53^. Since Rac1 activity plays a crucial role in epithelial cell-cell adhesion, basement membrane deposition, and protrusion^54^,^55^, further investigation of type I collagen-dependent Rac1 signaling in driving epithelial phenotype is warranted.

The collagen-induced mesenchymal shift we observe here is important because it directly illustrates an ECM-mediated destabilization of the mammary epithelial phenotype and suggests that the acquisition of mesenchymal gene expression by exposure to type I collagen could enable epithelial cells to then invade autonomously (if they were led into the stroma in the first place) or exacerbate existing basement membrane defects (if focal loss of basement membrane exposed cells to stromal ECM). In support of this notion, transient and localized loss of basement membrane at the invasive front is correlated with increased metastasis and poor patient survival^56^, indicating there need not be a widespread dissolution of basement membrane for invasion. Based on our results that collagen I upregulates expression and deposition of fibronectin, which promotes its own expression in mammary epithelium^49^, and MT1-MMP, which functions broadly in the proteolytic processing of ECM, cell surface, and soluble substrates^57^, it is therefore tempting to speculate that a collagen-induced mesenchymal shift could initiate feed-forward mechanisms to drive tumor invasion. Although previous work has reported matrix-dependent migration phenotypes in Matrigel and type I collagen in the absence of any EMT-like molecular response to collagen^25^, this study used RNA that was extracted from whole cultures including large numbers of cells within the organoid interior, so molecular changes in the cells directly contacting ECM may not have been detectable. In contrast, all organoids start as single cells contacting the ECM in our model, allowing us to detect differential matrix-dependent gene expression. Of note, we have previously reported that multicellular spheroids comprised of MCF-10A cells alone remain non-invasive when embedded within the same 3D collagen matrices that induce mesenchymal traits in MCF-10A cells in the present study^8^. However, there are several key differences between the two models that explain the apparently contradictory invasive and non-invasive MCF-10A behaviors observed in this and our previous work, respectively. In the multicellular spheroid model, 5 × 10^3^ cells are cultured in Matrigel-containing media for 48 h to form spheroids that are then embedded within collagen. Although we did not further characterize MCF-10A epithelial spheroids or test for a cell-derived basement membrane, spheroid morphologies suggested strong cell-cell cohesion, and we cannot rule out the involvement of basement membrane components from the Matrigel maintaining non-invasive epithelial behavior in collagen. Here, isolated MCF-10A cells were exposed to defined 3D ECM immediately and the resulting gene expression and phenotypic responses were characterized. In light of these findings, future work should use MCF-10A as well as additional cell models to define the molecular mechanisms through which basement membrane and stromal ECM drive epithelial and mesenchymal phenotypes, respectively.

MT1-MMP is a membrane-tethered proteinase whose substrates include ECM components, cell surface receptors, and other MMPs^57^. Notably, MT1-MMP has been shown to serve both pericellular collagenolytic and other roles during epithelial branching^31^,^58^ as well as collagen-invasive migration of epithelial cells^59^. MT1-MMP was recently identified as a major regulator of the transition from *in situ* to invasive carcinoma^60^, is upregulated at the invasive front of tumor cells^30^, and is required for blood vessel invasion and metastasis of breast cancer cells^61^. Here, we show that MT1-MMP expression is induced by 3D collagen and suppressed by 3D Matrigel in nontransformed mammary epithelial cells, and demonstrate that MT1-MMP activity is required for outward cell body translation and the formation of nucleus-containing protrusions in stroma-like ECM. Type I collagen-dependent MT1-MMP upregulation has been observed in normal and tumor cells^62^,^63^,^64^ and is suspected to occur via β1-integrin-dependent Src signaling that upregulates the transcription factor Egr-1 to promote MT1-MMP expression. Since MT1-MMP expression has also been shown to be controlled by MEK/ERK and PI3K/Akt signaling in different cell types and under varying stimuli^65^,^66^, the mechanisms by which type I collagen promotes MT1-MMP upregulation in mammary epithelial cells remain to be determined.

Here, we found that MT1-MMP is only required for invasion through small collagen matrix pores. These results contrast with work by Mori *et al*. indicating that MT1-MMP knockdown prevents epithelial branching morphogenesis in both dense and sparse collagen matrix^31^. However, while organoid formation is proliferation-dependent in our model, multicellular epithelial aggregates are formed outside matrix and then embedded in collagen in the model employed by Mori *et al*. Notably, we found that more extensive MT1-MMP knockdown or inhibition with GM6001 prevents cell proliferation and increases cell death after 4 days of 3D culture (data not shown), consistent with previous work showing that MT1-MMP activity is required for 3D cell growth^67^. Together, these results suggest that a relatively modest knockdown may leave sufficient MT1-MMP remaining to accomplish its non-proteolytic functions including integrin signaling that are required for invasion and branching in sparse collagen matrix^31^. Since the catalytic activity of MT1-MMP appears to be a rate-limiting requirement for proliferation and invasive outgrowth of epithelial cells in restrictive 3D collagen matrix, future work should explore the transcriptional, translational, and post-translational mechanisms by which specific mammary epithelial extracellular matrices regulate MT1-MMP^68^,^69^,^70^,^71^,^72^.

Mammary epithelial cell phenotype is dependent upon the mechanical properties of the ECM microenvironment^23^,^40^,^73^,^74^. We show here that 3D collagen matrix mechanics play a role in determining whether epithelial cells acquire an invasive epithelial phenotype as well as the nature of that phenotype. We first demonstrate by floating collagen matrices that intra-matrix tension is a requirement for the invasive epithelial phenotype, which unto itself is not surprising as cell-matrix mechanocoupling is a central determinant of epithelial phenotype^40^. Interestingly, inhibition of cell contractility via ROCK inhibition overcomes this suppression and promotes protrusion to rescue the invasive epithelial phenotype. This finding suggests that the potential for invasion is maintained in floating matrix, but is suppressed by ROCK activity when matrix tension is released. Indeed, we found that MCF-10A cells deposit fibronectin and not laminin in floating collagen matrix, which is consistent with ECM deposition in attached collagen matrix and suggests that collagen ECM provides signaling to drive mesenchymal gene expression independently of matrix anchorage. Of particular relevance to our results, it was recently reported that relaxation of myosin-II contractility weakens cell-cell cohesion^73^ and enhances Rac1 activity in mammary epithelial cells^75^. These results suggest that epithelial cells in our model may generate enhanced contractility to stabilize cell-cell adhesions in soft floating matrix, which restricts Rac1-dependent invasion. Thus, ROCK inhibition might serve to destabilize cell-cell adhesion and reactivate Rac1 to promote protrusive invasion, which is consistent with a release of cortical tension leading to protrusive activity^76^.

Interestingly, although we show here that Rac1 is required for the formation of protrusive structures in 3D type I collagen, it has also been shown that Rac1 activity downstream of β1-integrin adhesion regulates mammary epithelium polarity^35^ and differentiation^77^ in basement membrane culture. These seemingly opposing roles for Rac1 in mediating epithelial phenotype suggest that ECM composition might modulate signaling upstream and/or downstream of Rac1 to promote distinct matrix-dependent cell behaviors. Notably, Chaudhuri *et al*. demonstrated that constitutive activation of Rac1 in soft basement membrane ECM promotes a protrusive malignant phenotype while suppression of Rac1 activity prevents invasive behavior in stiff basement membrane ECM^21^. Together, these results indicate that matrix mechanics and composition provide critical signaling inputs that ultimately define the effect of Rac1 signaling on mammary epithelial cell phenotype. Considering recent findings in mammary epithelial cells that type I collagen enhances Rho activity as compared to Matrigel, and matrix-dependent Rac1 activity can be tuned through regulation of Rho activity^75^, the ECM context-dependent expression and activation of Rho GTPases is of particular interest.

We found that increasing 3D collagen matrix stiffness via non-enzymatic glycation promotes an increasingly dispersive invasive phenotype in mammary epithelial cells. While we did not examine the mechanisms by which enhanced 3D matrix stiffness downregulates cell-cell cohesion to promote epithelial dispersion here, it is likely that Rho GTPases play a significant role in this behavior^23^,^40^,^55^,^78^, and these mechanisms are currently under investigation. Our finding that increasing 3D ECM stiffness alone is sufficient to induce significant phenotypic changes in epithelial cells is in contrast to work in which both matrix stiffening and oncogene induction were required to drive invasive behavior^23^. However, there are considerable experimental differences including ECM composition and the method and extent of collagen crosslinking that likely can explain the observed differences^23^. Nonetheless, these results suggest that additional “hits” (e.g., oncogene induction) may be required to fully drive invasive epithelial behavior in response to smaller increases in ECM stiffness and/or in the presence of an established basement membrane. These findings further indicate that ECM composition and mechanics co-regulate mammary epithelial phenotype, and imply that future studies should continue to explore this relationship more deeply.

Finally, the distinct matrix-dependent gene expression patterns in Matrigel and collagen as well as the intermediate phenotypes observed in mixed Matrigel/collagen culture suggest that basement membrane and type I collagen may stimulate opposing cell signaling and phenotypic responses in epithelial cells. Indeed, specific integrin-ligand combinations can lead to distinct signaling and phenotypic outcomes^79^, and ECM composition can regulate integrin expression and signaling in mammary epithelial cells^16^,^80^. Especially since complete basement membrane dissolution is not necessary for invasion and cells can re-express basement membrane at secondary sites during metastasis^56^, it is conceivable that epithelial/carcinoma cells perceive mixed ECM signals during both early epithelial tissue destabilization and later metastatic seeding. However, the extent to which basement membrane protects and type I collagen deregulates normal epithelial cell phenotype remains unclear. Because of the close association among ECM biochemistry, mechanics, and structure, an effective strategy to describe this complex biology could exploit synthetic matrices that precisely emulate native stromal tissue, allow for the experimental isolation of ECM effects, and overcome co-variance of ECM features that can confound results from studies using natural matrices^21^. Such future work using rationally designed composite ECM models to identify the relative contributions of basement membrane and collagen I in regulating epithelial signaling and phenotype would provide further insight to our findings here as well as to both developmental and tumorigenic epithelial cell processes in general.

## Methods

### Cell culture and reagents

MCF-10A mammary epithelial cells (CRL-10317; ATCC, Rockville, MD) and MCF-10A stably expressing GFP-tagged H2B (MCF-10A/H2B-GFP; kind gift from Warren Zipfel, Cornell University, Ithaca, NY) were maintained in DMEM:F12 medium (Life Technologies, Grand Island, NY) supplemented with 5% horse serum, 20 ng/ml hEGF, 100 U/ml penicillin, and 100 μg/ml streptomycin (all from Life Technologies), 0.5 μg/ml hydrocortisone, 10 μg/ml insulin, and 100 ng/ml cholera toxin (all from Sigma-Aldrich, St. Louis, MO). MCF-10A growth medium containing 5 ng/ml hEGF was used for 3D cultures. All cell culture and time-lapse imaging was performed at 37 °C and 5% CO_2_.

The following reagents were used for fluorescent imaging: anti-E-cadherin antibody (sc-7870), anti-laminin γ2 antibody (sc-25341), and anti-fibronectin antibody (sc-80559) from Santa Cruz Biotechnology, Santa Cruz, CA; anti-vimentin antibody (V6389; Sigma-Aldrich); Alexa Fluor 568-conjugated anti-rabbit secondary antibody (A-11036), Alexa Fluor 568-conjugated anti-mouse secondary antibody (A-11004), Alexa Fluor 488-conjugated phalloidin, and Alexa Fluor 568-conjugated phalloidin from Life Technologies; DAPI (Sigma-Aldrich). The broad spectrum MMP inhibitor GM6001 (0.1 μM; EMD Millipore, Billerica, MA), Rac1 inhibitor NSC23766 (25 μM; Santa Cruz Biotechnology), Src kinase inhibitor PP1 (5 μM; Sigma-Aldrich), PI3K inhibitor LY294002 (10 μM; EMD Millipore), and ROCK inhibitor Y27632 (10 μM; Beckman Coulter, Brea, CA) were used as described below.

For MT1-MMP knockdown experiments, cells were transfected with 1 nM MT1-MMP siRNA or control siRNA (Table 1) using Lipofectamine 2000 (2 μg/ml; Life Technologies). Medium was changed following 4 h of transfection and cells were seeded for experiments the following day.

### 3D epithelial morphogenesis culture

MCF-10A cells were seeded in 3D ECM gels and cultured for 4 days to monitor matrix-dependent epithelial morphogenesis. Matrigel (growth factor-reduced, Corning Life Sciences, Lowell, MA) was diluted with 3D culture medium to a final matrix concentration of 4 mg/ml. Three-dimensional collagen matrix was prepared from acid-extracted rat tail tendon type I collagen as previously described^81^. Briefly, 10 mg/ml collagen stock was diluted with 3D culture medium to final matrix concentrations of 1.0 mg/ml or 1.5 mg/ml and neutralized with 1 M sodium hydroxide. Mixed collagen/Matrigel matrices were prepared using the above protocols to generate final concentrations of each component as indicated. Non-enzymatic glycation of collagen was performed as previously described^38^. Collagen stock solution was mixed with 0.5 M ribose in 0.1% acetic acid to form glycated collagen solutions containing a final concentration of 0, 50, or 100 mM ribose and incubated at 4 °C for 5 days. Glycated collagen solutions were buffered to final concentrations of 25 mM HEPES and 44 mM sodium bicarbonate, and neutralized with 1 M sodium hydroxide to form 1.5 mg/ml collagen gels. Cells were incorporated into diluted Matrigel or neutralized collagen solutions at 10^5^ cells/ml and solutions were polymerized at 37 °C before being overlaid with 3D culture medium, which was changed, with or without inhibitors, every other day during experiments. For 2D gene expression control, cells were seeded on type I collagen-coated (0.1 mg/ml) glass.

### Quantitative Real-Time RT-PCR

Total RNA was extracted using the RNeasy Mini Kit (Qiagen, Valencia, CA) from cell pellets trypsinized from 2D tissue culture as indicated or from 3D samples following matrix digestion with collagenase type 4 (Worthington Biochemical, Lakewood, NJ) or collagenase/dispase (Roche, Indianapolis, IN) per manufacturer instructions. RNA was reverse transcribed into cNDA using qScript cDNA SuperMix (Quanta Biosciences, Gaithersburg, MD) according to manufacturer’s instructions and quantitative RT-PCR was performed using SYBR Green SuperMix (Quanta Biosciences) and an iCycler IQ Real-Time PCR detection system (Bio-Rad Laboratories, Hercules, CA). Gene expression was normalized to GAPDH. Custom primers were obtained from Life Technologies and are listed in Table 1.

### Indirect immunofluorescence and imaging

For immunofluorescence imaging, 3D matrix samples were fixed with buffered 3.7% formaldehyde, rinsed, permeabilized with 0.1% Triton X-100, and blocked with bovine serum albumin (1% w/v) and 10% fetal bovine serum (10% v/v) in PBS before overnight incubation with primary antibody in blocking solution (1:50). Samples were washed extensively before overnight incubation with secondary antibody in blocking solution (1:100). Fixed samples were labelled with phalloidin and DAPI for organoid morphology characterization. All imaging was performed using a Zeiss LSM700 confocal scanning head on a Zeiss Axio Observer Z1 inverted microscope (Carl Zeiss Microscopy, Thornwood, NY). A ×40 long working distance water immersion lens was used for immunofluorescence and collagen matrix imaging by confocal reflectance microscopy, and a ×10 lens was used for organoid morphology and time-lapse imaging. Brightfield images were acquired by detection of transmitted laser light using a T-PMT. Collagen matrix structure was quantified by manual measurement of matrix pore cross sectional areas from single confocal reflectance image slices of matrix immediately following polymerization. Epithelial organoid morphology was quantified by measurement of organoid cross sectional area, circularity (4π **·** (area/perimeter^2^)), and protrusive structure lengths. Organoid circularity was used to categorize organoid morphology: acinar (round, non-invasive colonies; circularity >0.8); protrusive acinar (acinar colonies with protrusions; circularity 0.6–0.8); invasive (protrusive and stellate colonies; organoid circularity <0.6). All image analysis was performed using ImageJ (version 1.49b, National Institutes of Health, Bethesda, MD).

### Statistical analysis

Data are presented as mean ± s.e.m., or box-and-whisker plots, where boxes represent medians and 25^th^/75^th^ percentile and bars indicate the 5^th^ and 95^th^ percentiles. Means were compared by one-way analysis of variance (ANOVA) with a post hoc Tukey’s HSD test using JMP (version 10; SAS Institute, Cary, NC).

## Additional Information

**How to cite this article**: Carey, S. P. *et al*. Three-dimensional collagen matrix induces a mechanosensitive invasive epithelial phenotype. *Sci. Rep.* **7**, 42088; doi: 10.1038/srep42088 (2017).

**Publisher's note:** Springer Nature remains neutral with regard to jurisdictional claims in published maps and institutional affiliations.

## Figures and Tables

**Table 1:** Nucleotide sequences for siRNA and qRT-PCR.

**siRNA primers**	
MT1-MMP siRNA (NM_004995.2)	5′-CCUACGAGAGGAAGGAUGGCAAAUU-3′
Control siRNA	5′-UUCCUCUCCACGCGCAGUACAUUUA-3′
**Primers for qRT-PCR**	
E-cadherin	5′-TTGACGCCGAGAGCTACA-3′
	5′-GACCGGTGCAATCTTCAAA-3′
N-cadherin	5′-ACCAGGTTTGGAATGGGACAG-3′
	5′-ATGTTGGGTGAAGGGGTGCTTG-3′
Vimentin	5′-TGAAGGAGGAAATGGCTCGTC-3′
	5′-GTTTGGAAGAGGCAGAGAAATCC-3′
Snail	5′-ATCGGAAGCCTAACTACAGCGAGC-3′
	5′-CAGAGTCCCAGATGAGCATTGG-3′
Fibronectin	5′-GATAAATCAACAGTGGGAGC-3′
	5′-CCCAGATCATGGAGTCTTTA-3′
MT1-MMP	5′-CGCTACGCCATCCAGGGTCTCAAA-3′
	5′-CGGTCATCATCGGGCAGCACAAAA-3′
GAPDH	5′-CATGAGAAGTATGACAACAGCCT-3′
	5′-AGTCCTTCCACGATACCAAAGT-3′
